# Relationship between gout, hyperuricemia, and obesity—does central obesity play a significant role?—a study based on the NHANES database

**DOI:** 10.1186/s13098-024-01268-1

**Published:** 2024-01-22

**Authors:** Tongjun Mao, Qian He, Junping Yang, Lanlan Jia, Guofei Xu

**Affiliations:** 1 Department of Rheumatology, The First Affiliated Hospital of Wannan Medical College, Yijishan Hospital of Wannan Medical College, Wuhu, China; 2 Anhui Normal University School of Educational Sciences, Wuhu, China; 3 Department of General Practice, Wuhu City SecondPeoplès Hospital, Wuhu, Anhui China

**Keywords:** Hyperuricemia, Gout, Cross-sectional study, BMI,WWI and BRI, Central obesity

## Abstract

**Background:**

Our objective was to evaluate how various measures of obesity, such as body mass index(BMI), body roundness index(BRI), and weigh adjusted waist index(WWI), influence urate levels, prevalence of gout and to compare the disparities among these obesity indicators.

**Methods:**

By analyzing the 2001–2018 National Health and Nutrition Examination Survey (NHANES), we assessed the relationship between BMI, WWI, and BRI indices and urate levels, hyperuricemia, and the prevalence of gout. Smoothed curve fitting was used to determine whether there was a nonlinear relationship between BMI,WWI, and BRI indices and urate levels, hyperuricemia, and the prevalence of gout, and threshold effects analysis was used to test this relationship. We also used ROC curves to determine the diagnostic efficacy of BMI, WWI, and BRI on the prevalence of hyperuricemia and gout.

**Results:**

The study incorporated a total of 29,310 participants aged over 20 years, out of which 14,268 were male. Following the adjustment for the pertinent confounding factors, it was observed that higher levels of BMI, WWI, and BRI were significantly associated with a gradual and dose-dependent increase in urate levels. In the sensitivity analysis, each unit increment in BMI, WWI, and BRI levels exhibited an 8%, 72%, and 26% respective elevation in the risk of hyperuricemia, as well as a 5%, 31%, and 15% respective increase in the risk of gout. Dose-response curves provided evidence of a linear positive correlation between BMI, WWI, BRI, and urate levels, as well as the prevalence of hyperuricemia and gout. Based on the response from the ROC curve, overall, the diagnostic efficacy of BRI for hyperuricemia and gout surpasses that of BMI.

**Conclusion:**

The central obesity indices WWI and BRI levels are superior to BMI in detecting the prevalence of urate levels, hyperuricemia, and gout, and although a clear causal relationship has not yet been established, it is important to recognize the impact of central obesity on uric acid levels and to give it due attention.

**Supplementary Information:**

The online version contains supplementary material available at 10.1186/s13098-024-01268-1.

## Introduction

Urate represents the final outcome of purine metabolism. Hyperuricemia is distinguished by the presence of abnormally elevated concentrations of urate in the bloodstream and is recognized as an antecedent to the development of gout. Gout, characterized as a clinical manifestation arising from the deposition of crystalline monosodium urate (MSU), stands as the prevailing form of inflammatory arthritis in the adult population. It affects an estimated global populace of approximately 41 million individuals, with its prevalence showing an upward trend worldwide [1]. Notably, while hyperuricemia is more prevalent, it should be distinguished from the distinct condition of gout [2]. Despite a comprehensive understanding of the disease’s underlying mechanisms and the availability of treatments, the burden of gout continues to be significant, and the management of gout falls short of optimal standards [3]. Patients with gout commonly experience a combination of various underlying health conditions, such as hypertension, chronic kidney disease (CKD), obesity, and cardiovascular disease (CVD). These conditions, when present alongside gout, further exacerbate the burden of the disease and are associated with a higher risk of morbidity and mortality [4]. Moreover, patients with gout have an elevated incidence of metabolic syndrome, which increases the likelihood of developing “type 2” diabetes [5, 6], CKD [7], aortic stenosis [8], end-stage renal failure [9], ischemic stroke, and peripheral vascular disease [10]. Hyperuricemia is also linked to an increased frequency of cardiovascular death [11], coronary heart disease [12], heart failure, atrial fibrillation [13], and stroke [14]. It can lead to hypertension [15] and, when associated with hyperuricemia [16, 17], renal failure [18], type 2 diabetes [19], and metabolic syndrome [20], treating hypertension becomes more challenging. Therefore, it is crucial to comprehend the risk factors contributing to the development of gout and hyperuricemia in order to prevent and reduce costs associated with these conditions.

Obesity represents a significant global health challenge, with a staggering number of 1.9 billion adults reported to be overweight or obese [21], and this number continues to rise. Obesity stands as a primary contributor to numerous life-threatening diseases, including type II diabetes, hypertension, stroke, myocardial infarction, sleep apnea, and heart disease. It is a complex chronic disease influenced by various factors such as genetics, behavior, diet, socioeconomic status, and environment [22]. Multiple studies have consistently demonstrated the association between obesity and an increased risk of hyperuricemia and gout [23]. Weight has consistently been identified as a key determinant of serum urate levels [24], and weight loss has the potential to mitigate the risk of developing gout [25]. A study conducted in 2019 involving over 5,000 patients revealed that three months after undergoing bariatric surgery, the mean decrease in serum urate levels was 0.73 mg/dL, and this reduction continued to be 1.91 mg/dL at the three-year postoperative mark [26]. Furthermore, the prevalence of abdominal obesity is higher among gout patients compared to those without gout [27], and the risk of gout increases with obesity [25].

When it comes to defining obesity, the most commonly utilized anthropometric measure in clinical and epidemiological studies is body mass index (BMI) [28–30]. The World Health Organization (WHO) also recommends the use of BMI for defining obesity and overweight [31]. However, in recent years, there have been the emergence of new obesity indices. Two such indices are the body roundness index (BRI) [32] and the weight-adjusted waist circumference index (WWI) [33]. BRI and WWI primarily assess central obesity, similar to BMI, but from a different perspective, reflecting the level of obesity in the body. It has been demonstrated that central obesity exhibits a stronger association with insulin resistance, diabetes, and cardiovascular disease (CVD) compared to general obesity [34].

However, in the context of hyperuricemia and gout, the majority of published clinical literature tends to focus on obesity alone, neglecting the attention towards individuals who may not be generally obese but exhibit central obesity. Anthropometry is a widely utilized, cost-effective, and straightforward technique. It holds crucial clinical and public health implications to identify the anthropometric indicators that are most strongly associated with elevated urate levels, hyperuricemia, and gout.

This study presents a nationally representative survey employing data from adult participants in the NHANES survey spanning from 2007 to 2018. The objective of this study was to conduct a detailed assessment of the role and relationship of various obesity indices in evaluating urate levels and gout. We compared the correlation between different anthropometric indices in terms of baseline measurements and changes over time, as well as the disparity between BMI and central obesity indices regarding their impact on urate levels and incidence of gout. This study stands as the first to distinguish the potential correlation between general obesity and central obesity with an increased risk of elevated urate levels, hyperuricemia, and the prevalence of gout.

## Materials and methods

### Study population

For this study, we chose to analyze data from six survey cycles of NHANES, covering the years 2007 to 2018, in a cross-sectional manner. 59,842 people completed the survey. The NHANES survey employs a complex multi-stage sampling technique to gather its data. It covers various aspects, including sociodemographics, dietary intake, health behaviors, medical history, physiology, and laboratory tests. All protocols of NHANES strictly adhere to the U.S. Department of Health and Human Services (HHS) Human Research Subject Protection Policy. Ethical approval for this study was obtained from the National Center for Health Statistics (NCHS), and all participants provided both oral and written consent. It is important to note that all data used in this study were made available by NHANES free of charge and did not require additional authorization or ethical review.

### Data collection and definition

The body roundness index (BRI) serves as an exposure variable and is calculated using the formula BRI = 364.2-365.5 × {1 - [(WC/2π)/(0.5 × height)] ^2^ } ^0.5^(WC = waist circumference). The BMI is calculated as the individual’s weight in kilograms divided by the square of their height in meters. For each participant, the WWI is determined by taking the square root of the waist circumference in centimeters divided by the weight in kilograms. Skilled examiners at the mobile examination center obtained basic anthropometric measurements, including weight, height, and waist circumference, using standardized techniques and equipment. urate concentrations were measured using the Beckman Unicel DxC 800 Synchron Clinical System. Hyperuricemia was defined as a serum urate concentration of 7.0 mg/dL or higher in men and 6.0 mg/dL or higher in women [35]. Given that urate (MSU) crystallizes after exceeding 7 mg/dL, potentially advancing to gout, we have established this threshold as the critical value for hyperuricemia. In our analysis, we conducted a sensitivity analysis to explore the impact of various obesity indices on the occurrence of hyperuricemia.Questionnaires were also administered to collect information on the presence or absence of gout(participants who answered “yes” explicitly were recognized as having gout), and serum urate, hyperuricemia, and gout occurrence were designated as the outcome variables in the study.

We considered several potential covariates that could influence the relationship between the obesity index and urate levels, and these were accounted for in a multivariate adjusted model. The covariates included sex, age, race, education level, poverty to income ratio (PIR), marital status, alcohol consumption, physical activity level, cholesterol level (mg/dL), triglyceride level (mg/dL), fasting glucose level (mg/dL), smoking status, presence of hypertension, diabetes, coronary heart disease, cancer, and various dietary intake factors such as energy intake, fat intake, sugar intake, and water intake. Detailed information on the measurement procedures for these study variables can be found on the public website www.cdc.gov/nchs/nhanes.

Treatment of missing values: A few numerical variables in our data include a high number of missing values. To solve this problem, we transformed these variables into categorical variables and represented the missing values as independent sets of dummy variables.

### Statistical methods

We utilized the provided sampling weights, stratification, and clustering from the NHANES study to account for the complex, multistage sampling design used in selecting a representative noninstitutionalized U.S. population. Continuous variables were presented as weighted survey means with 95% confidence intervals, while categorical variables were presented as weighted surveys with 95% confidence intervals. To examine the relationship between the obesity index and urate, we employed linear regression analysis according to the guidelines. For assessing the association between the obesity index and hyperuricemia and gout, we utilized multivariate logistic regression analysis. To evaluate the independent effects of the covariates on the dependent variables, we generated three models with different adjusted covariates. Model 1 involved no adjustment for covariates, Model 2 included adjustment for age, sex, race, marriage, and education level, and Model 3 encompassed adjustment for all covariates listed in Table 1. We performed generalized weighted smoothed curve fitting analysis to determine if there existed a nonlinear relationship between obesity and urate levels. This analysis was further validated through threshold effect analysis to identify significant inflection points. In threshold effect analysis, a log-likelihood ratio (LLR) less than 0.05 indicated a nonlinear correlation between the independent and dependent variables. To assess the diagnostic efficacy of BMI, WWI, and BRI in predicting hyperuricemia and gout, we employed dichotomous ROC curve analysis. A p-value of less than 0.05 was considered statistically significant, indicating a significant difference.

## Results

### General participant characteristics

**Fig. 1:**
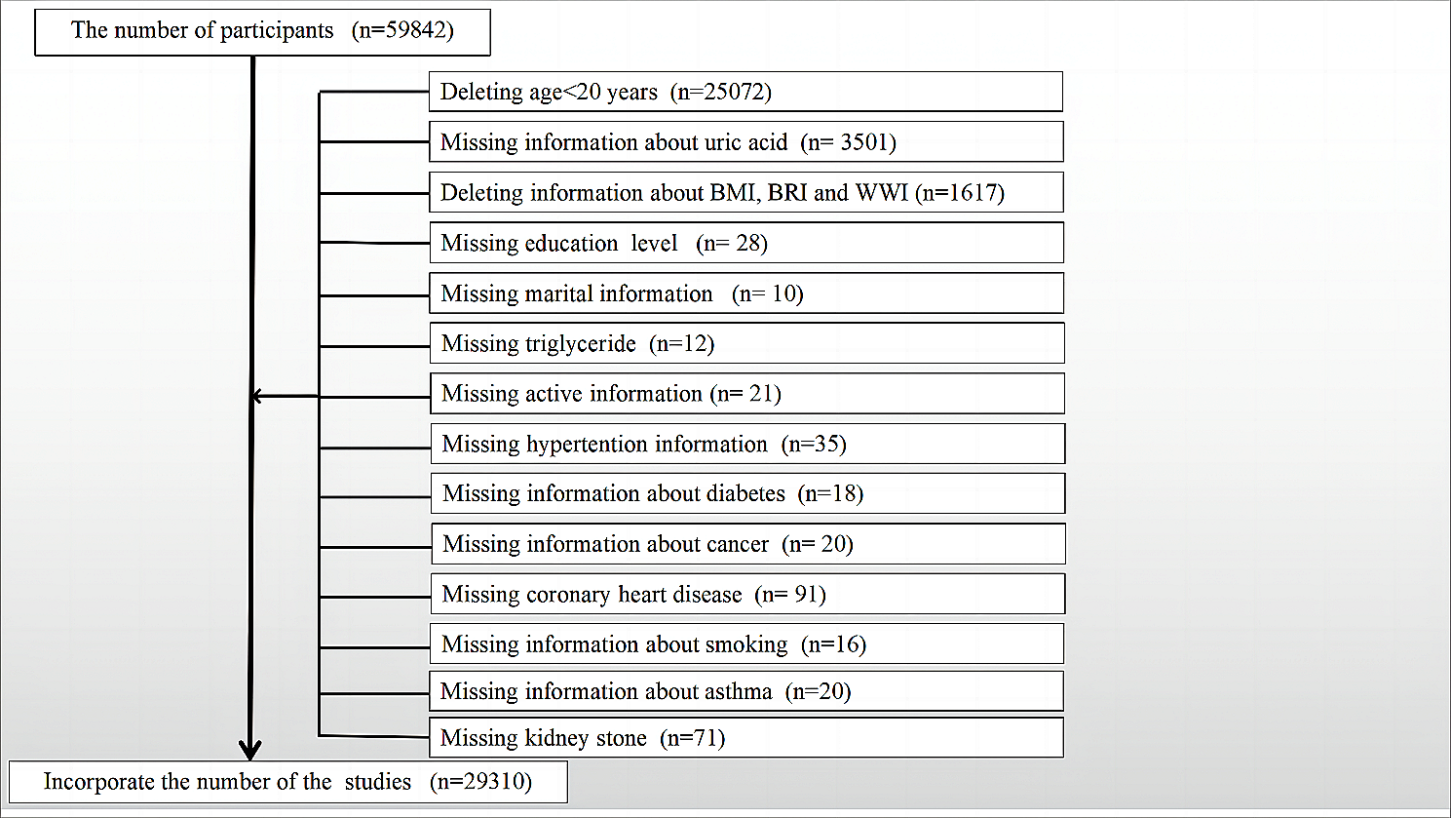
Flow chart for participants

The demographic characteristics of the participants included in this study are presented in Table 1. Figure 1 illustrates the population screening process. Specifically, we first excluded minors under 20 years of age (*n* = 25,072) because the survey only involved adults. We then excluded participants with missing urate (*n* = 3501) and missing BMI/BRI/WWI index (*n* = 1617). We also excluded missing information on education level, marital status, triglycerides, physical activity, and smoking (*n* = 87). For self-report, we excluded participants with missing information on several diseases such as hypertension, diabetes, coronary heart disease, asthma, cancer, and kidney stones (*n* = 255). After applying these exclusion criteria, we ended up with a study sample of 29,310 participants, which included 5,882 participants with hyperuricemia and 1,324 participants with self-reported gout. The weighted characteristics were divided into quartiles based on urate levels: Q1 (0.4–4.4 mg/dL), Q2 (4.4–5.3 mg/dL), Q3 (5.3–7.3 mg/dL), and Q4 (7.3–18 mg/dL). Baseline characteristics showed significant differences among the urate quartiles, except for marital status. Individuals in the highest urate quartiles tended to be older and had higher levels of cholesterol, triglycerides, fasting glucose, BMI, BRI, WWI, a higher prevalence of kidney stones, diabetes, hypertension, coronary heart disease, and a higher proportion of alcohol and tobacco users.

### Relationship between obesity-related indices and urate levels

**Fig. 2:**
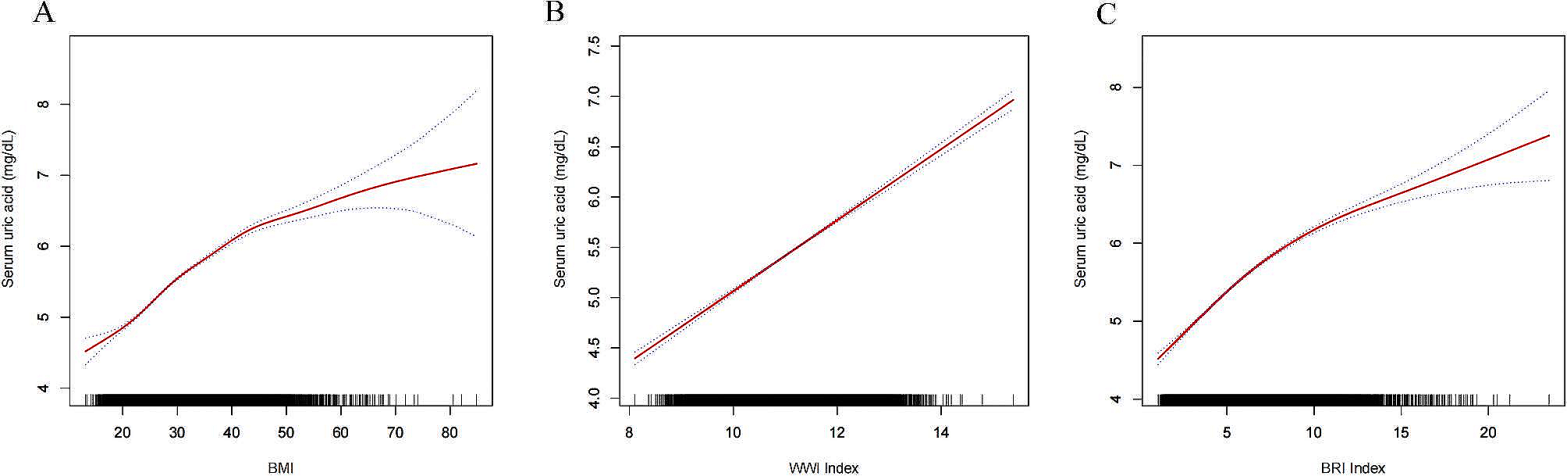
A. The graph depicts the density dose-response relationship between BMI and serum urate levels. B. The graph illustrates the density dose-response relationship between WWI and serum urate levels. C. The graph showcases the density dose-response relationship between BRI and serum urate levels. The shaded area between the upper and lower dashed lines represents the 95% confidence interval. Each data point represents the magnitude of the index and is connected to form a continuous line. The analysis was adjusted for all covariates except the effect modifier

We used multiple linear regression analysis to examine the relationship between obesity-related indices and urate levels. The results, presented in Table 2, demonstrate that in the fully adjusted model, elevated BMI (β = 0.06, 95% CI: 0.05, 0.06), WWI (β = 0.35, 95% CI: 0.33, 0.37), and BRI (β = 0.17, 95% CI: 0.16, 0.17) were positively associated with higher urate levels. As BMI, WWI, and BRI increased, urate levels exhibited a significant and gradual increase in a dose-dependent manner (p for trend < 0.01). To further investigate this association, we conducted fitted curve analysis, which confirmed a dose-response relationship between the three obesity markers and urate levels. The analysis also revealed that the association between the obesity markers and urate followed a linear positive correlation (Fig. 2).

### Association between obesity-related indices and hyperuricaemia and gout

**Fig. 3:**
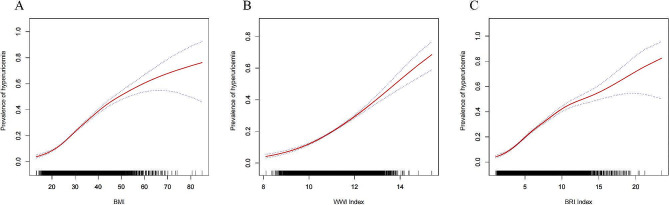
A. The graph demonstrates the density dose-response relationship between BMI and the prevalence of hyperuricemia. B. The graph depicts the density dose-response relationship between WWI and the prevalence of hyperuricemia. C. The graph showcases the density dose-response relationship between BRI and the prevalence of hyperuricemia. The shaded area between the upper and lower dashed lines represents the 95% confidence interval. Each data point represents the magnitude of the index and is connected to form a continuous line. The analysis was adjusted for all covariates except the effect modifier

**Fig. 4:**
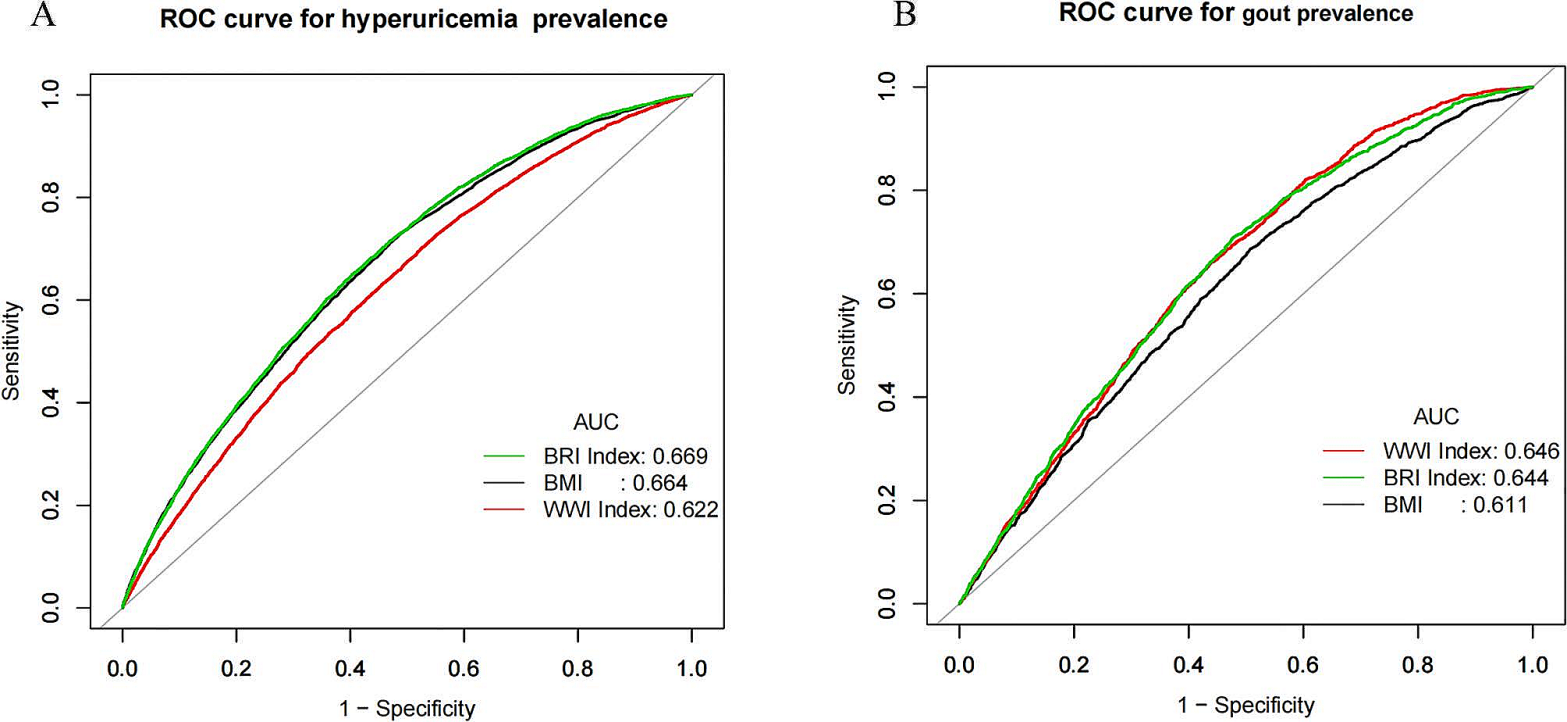
A. Diagnostic performance of obesity index for hyperuricemia prevalence. B. Diagnostic performance of obesity index for gout prevalence

Table 3 presents the results of logistic regression analyzing the impact of three obesity indices on the prevalence of hyperuricemia and gout. Regarding hyperuricemia, we observed a positive association between elevated BMI (OR = 1.08, 95% CI: 1.08, 1.09), WWI (OR = 1.72, 95% CI: 1.64, 1.80), BRI (OR = 1.26, 95% CI: 1.25, 1.28) and an increased prevalence of hyperuricemia. When hyperuricemia was defined with a cutoff value of 7 mg/dl for urate, logistic regression analysis of the effects of multiple obesity indices on hyperglycemia remained highly significant with essentially the same values (see Supplementary Table 1).Participants in the highest BMI tertile exhibited a 2.57-fold increase (OR = 3.57, 95% CI: 3.27, 3.88) in the prevalence of hyperuricemia compared to the lowest tertile. Similarly, individuals in the highest WWI tertile had a 1.45-fold higher risk (OR = 2.45, 95% CI: 2.24, 2.69) of hyperuricemia compared to the reference group. For those in the highest BRI tertile, there was a 2.77-fold increased risk (OR = 3.77, 95% CI: 3.45, 4.12) of developing hyperuricemia compared to the reference group. Smoothing curve fitting illustrated a linear positive correlation between BMI, WWI, BRI levels, and the prevalence of hyperuricemia (Fig. 3). Next, we obtained ROC curves to evaluate the diagnostic efficacy of the three obesity indices for hyperuricemia. The analysis indicated that BMI, WWI, and BRI all exhibited statistically significant diagnostic efficacy for hyperuricemia (AUC > 0.5, *P* < 0.05) using dichotomous logistic regression analysis ( Fig. 4a; Table 4). Furthermore, BRI displayed a higher area under the ROC curve than BMI (AUC = 0.669).The optimal cut-off values for BMI,WWI and BRI for hyperuricaemia were 27.54, 10.91 and 5.42 respectively(Table 4).

**Fig. 5:**
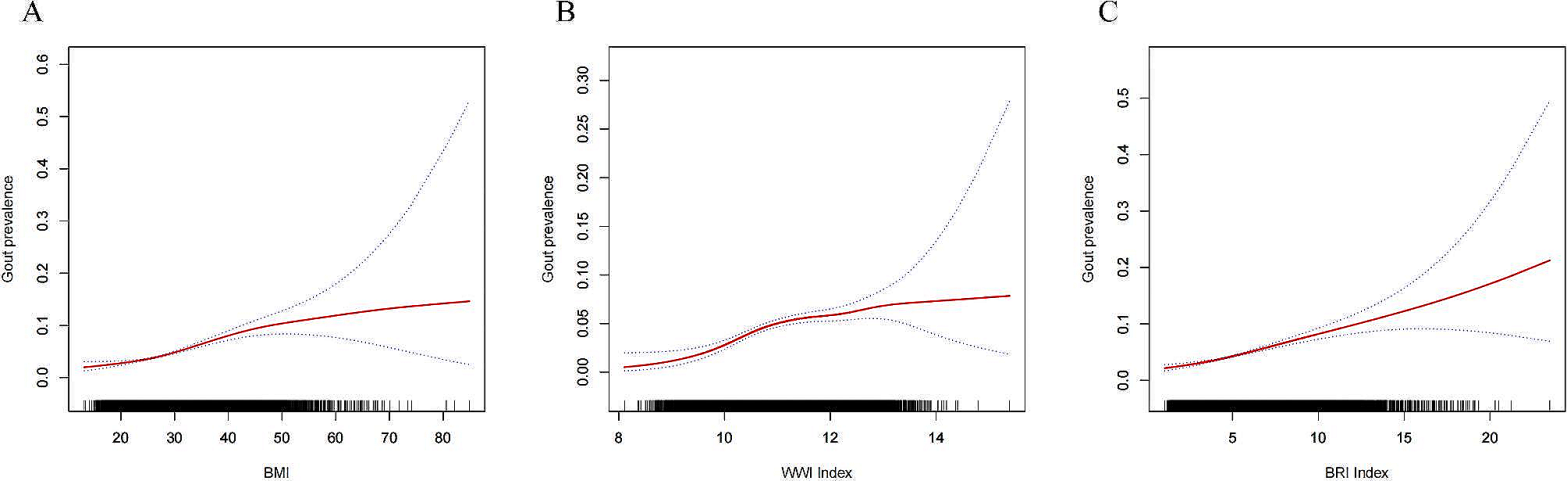
A. The graph illustrates the density dose-response relationship between BMI and the prevalence of gout. B. The graph displays the density dose-response relationship between WWI and the prevalence of gout. C. The graph demonstrates the density dose-response relationship between BRI and the prevalence of gout. The shaded area between the upper and lower dashed lines represents the 95% confidence interval. Each data point represents the magnitude of the index and is connected to form a continuous line. The analysis was adjusted for all covariates except the effect modifier

In the group with a history of gout, the fully adjusted model revealed a positive association between BMI, WWI, and BRI with gout prevalence. Each unit increase in BMI, WWI, and BRI was associated with a 5% (OR = 1.05, 95% CI: 1.04, 1.06), 31% (OR = 1.31, 95% CI: 1.19, 1.43), and 15% (OR = 1.15, 95% CI: 1.12, 1.18) increase in gout prevalence, respectively. The trend test further confirmed a dose-dependent effect of BMI, WWI, and BRI on the increased prevalence of gout (p for trend < 0.05), as shown in Table 5. Smoothing curve fitting illustrated a linear positive correlation between BMI, WWI, BRI levels, and gout prevalence (Fig. 5). After performing dichotomous logistic regression analysis, the results indicated that BMI, WWI, and BRI all obtained statistically significant diagnostic efficacy for gout (AUC > 0.5, *p* < 0.05) (Fig. 4b; Table 6). Moreover, WWI (AUC = 0.646) and BRI (AUC = 0.644) displayed a higher area under the ROC curve compared to BMI.The optimal thresholds for BMI, WWI and BRI for gout were 27.90, 11.19 and 5.22 according to Table 6.

## Discussion

This study is a comprehensive nationwide investigation examining the impact of various obesity indices on urate levels, hyperuricemia, and gout. Our findings reveal that BMI, BRI, and WWI exhibited positive associations with elevated urate levels, hyperuricemia, and gout incidence, respectively. Notably, BRI and WWI, which specifically measure central obesity, demonstrated higher sensitivity in predicting these conditions compared to BMI alone.

Obesity is a widespread global issue, affecting a significant number of adults worldwide, with over 1.9 billion individuals classified as overweight or obese (Obesity and overweight Factsheet). Numerous previous studies have convincingly demonstrated a strong correlation between obesity and the development of hyperuricemia and gout. For instance, higher body mass index (BMI) has been consistently linked to an elevated risk of hyperuricemia and gout [23], while weight loss has been shown to reduce the likelihood of developing gout [25]. Moreover, weight gain has been identified as a prominent factor associated with an excessive rise in serum urate levels [36]. Our study, consistent with previous research, once again highlights the significant role of obesity in the prevalence of elevated urate, hyperuricemia, and gout. Moreover, our study reveals an independent and positive correlation between obesity-related indices (BMI, BRI, and WWI) and the prevalence of hyperuricemia and gout in a dose-dependent manner. Further analysis using logistic regression indicates that BRI exhibits a higher area under the ROC curve compared to BMI in predicting hyperuricemia. Similarly, in the case of gout, WWI demonstrates higher area under the ROC curve compared to BMI. Based on some previous studies, we believe that such results should be correct. The study by Takahashi et al. revealed a positive correlation between visceral obesity, quantified by visceral fat area (VFA), and uric acid (UA) metabolism. Interestingly, VFA demonstrated a stronger association with increased UA levels than body mass index (BMI), implying a potentially detrimental impact of visceral obesity on UA levels [37]. In a similar vein, Matsuura et al. emphasized the influence of visceral fat accumulation on hyperuricemia, highlighting its superiority over BMI as a contributing factor [38]. Further supporting these findings, a Japanese cross-sectional study discovered that central obesity, particularly in normal-weight individuals with central obesity, exhibited associations with hyperuricemia in both men and women [39]. Huang et al.‘s research indicated that the waist-to-height ratio, an indicator of central obesity, outperformed BMI as an independent predictor of hyperuricemia [40]. These consistent outcomes were also evident in a cross-sectional study involving 699 Korean subjects with diabetes [41].While these studies align with the present study’s findings, it is crucial to note the necessity for additional clarification and exploration of the underlying mechanisms. Future multicenter prospective cohort studies can provide a more comprehensive understanding of the intricate relationships identified in these studies and offer valuable insights into the implications of visceral obesity on UA metabolism.

Limited reports currently exist regarding the impact of obesity on urate metabolism, and the precise underlying mechanisms remain unclear. Pathophysiologically, individuals with obesity exhibit an imbalance between calorie intake and energy expenditure, resulting in the excessive accumulation of abdominal and visceral fat. This heightened adiposity contributes to an augmented overall nucleic acid metabolism, subsequently fostering uric acid synthesis through purine metabolism [42]. Furthermore, obesity may induce aberrations in glomerular hemodynamics and provoke the overactivation of the renin-angiotensin-aldosterone system, potentially leading to obesity-associated nephropathy. Prolonged exposure to these effects may contribute to glomerular atherosclerosis, ultimately reducing renal uric acid excretion [43, 44]. Insulin resistance, a common consequence of obesity [45], can further complicate urate metabolism by influencing the renal excretion of uric acid [46, 47]. Additionally, certain adipocytokines associated with obesity, such as adiponectin and leptin, have been reported to correlate with the development of hyperuricemia [48, 49]. While these observations shed light on the potential links between obesity and urate metabolism, further research is warranted to elucidate the intricate mechanisms involved in this relationship.

Our study possesses several notable strengths. Firstly, the NHANES study protocol that we strictly adhered to addresses important considerations such as sample weighting, ensuring the generalizability of our findings to the broader US population. Additionally, our large sample size provides robustness to our results and allows for validation. However, it is important to acknowledge certain limitations in our study. Firstly, the study design was primarily cross-sectional, confining our analysis to investigating the correlation between three distinct obesity indices (BMI, BRI, and WWI) and the prevalence of elevated urate, hyperuricemia, and gout. We did not scrutinize additional obesity-related indices, nor did we establish causative links or delve into the underlying mechanisms. Secondly, we were unable to access information on the history of pertinent medication use, including medications with a potential urate-raising effect (such as diuretics) or a urate-lowering effect (such as allopurinol). This limitation could potentially impact the robustness and reliability of the results. Lastly, it is worth noting that our assessment of gout relied on a questionnaire, which introduces the possibility of recall bias.

## Conclusion

This study indicates a potential link between obesity and increased urate levels, hyperuricemia, and the prevalence of gout. It suggests that managing obesity, as evaluated through obesity indices, could have positive implications for overall physical health. Additionally, the findings suggest that central obesity, which encompasses more than just pure obesity, may provide valuable insights for the management of urate and gout. However, it is important to note that further studies are required to validate and confirm our findings.

**Table 1:** Weighted characteristics in male subjects based on serum urate quartiles

Serum urate (mg/dL)	Q1(0.4–4.4 )	Q2(4.4–5.3 )	Q3(5.3–7.3 )	Q4(7.3–18 )	P-value
Age(years)	45.14(44.59,45.69)	46.29 (45.65,46.94)	48.36 (47.73,48.98)	48.85 (48.30,49.40)	< 0.0001
Serum Cholesterol(mg/dl)	191.49 (190.10,192.87)	192.77 (191.21,194.33)	194.06 (192.36,195.76)	197.17 (195.70,198.64)	< 0.0001
Serum Glucose(mg/dl)	97.37 (96.33,98.41)	98.46 (97.35,99.57)	99.52 (98.67,100.38)	102.26 (101.33,103.18)	< 0.0001
Serum Triglycerides(mg/dl)	120.94 (117.50,124.39)	140.25 (136.06,144.44)	160.88 (155.57,166.20)	186.21 (181.80,190.63)	< 0.0001
BMI(kg/m^2^)	26.41 (26.20,26.62)	28.32 (28.08,28.55)	29.69 (29.46,29.93)	31.37 (31.12,31.62)	< 0.0001
WWI Index	10.85 (10.82,10.89)	10.94 (10.91,10.97)	11.00 (10.97,11.03)	11.11 (11.08,11.13)	< 0.0001
BRI Index	4.64 (4.57,4.71)	5.19 (5.11,5.28)	5.58 (5.49,5.66)	6.09 (6.00,6.19)	< 0.0001
Gender(%)					< 0.0001
Male	14.38 (13.20,15.64)	37.70 (36.08,39.36)	61.52 (60.28,62.75)	77.02 (75.80,78.19)	
Female	85.62 (84.36,86.80)	62.30 (60.64,63.92)	38.48 (37.25,39.72)	22.98 (21.81,24.20)	
Race(%)					< 0.0001
Mexican American	9.91 (8.18,11.96)	9.13 (7.63,10.88)	8.40 (7.00,10.06)	7.42 (6.07,9.05)	
White	72.06 (69.47,74.50)	71.80 (69.03,74.42)	73.58 (70.97,76.03)	73.28 (70.76,75.66)	
Black	10.47 (9.09,12.03)	10.59 (9.19,12.18)	10.01 (8.62,11.61)	11.39 (9.92,13.05)	
Other Race	7.57 (6.74,8.49)	8.48 (7.38,9.72)	8.01 (6.96,9.20)	7.90 (6.97,8.96)	
Education Level(%)					< 0.0001
Less than high school	15.14 (13.76,16.64)	15.61 (14.12,17.23)	15.83 (14.29,17.50)	15.50 (14.26,16.83)	
High school	20.44 (18.92,22.06)	21.90 (20.46,23.42)	23.86 (22.43,25.36)	24.94 (23.27,26.68)	
More than high school	64.41 (61.90,66.84)	62.49 (60.17,64.76)	60.31 (58.09,62.48)	59.56 (57.41,61.67)	
Marital Status(%)					0.1501
Cohabitation	64.72 (62.92,66.47)	62.66 (61.09,64.20)	64.01 (62.30,65.68)	64.55 (62.70,66.36)	
Solitude	35.28 (33.53,37.08)	37.34 (35.80,38.91)	35.99 (34.32,37.70)	35.45 (33.64,37.30)	
Kidney Stone (%)					< 0.0001
No	91.73 (90.90,92.50)	91.07 (90.01,92.02)	89.34 (88.28,90.32)	88.53 (87.59,89.42)	
Yes	8.27 (7.50,9.10)	8.93 (7.98,9.99)	10.66 (9.68,11.72)	11.47 (10.58,12.41)	
Alcohol(%)					< 0.0001
Yes	55.02 (52.87,57.15)	58.95 (56.62,61.23)	63.65 (61.71,65.55)	65.99 (64.32,67.61)	
No	21.54 (20.01,23.16)	20.05 (18.46,21.74)	17.24 (15.89,18.69)	15.83 (14.52,17.23)	
Unclear	23.44 (21.59,25.40)	21.00 (19.10,23.05)	19.11 (17.55,20.76)	18.18 (17.00,19.43)	
High Blood Pressure (%)					< 0.0001
Yes	21.32 (19.88,22.83)	26.69 (25.23,28.20)	32.63 (30.91,34.40)	43.54 (41.73,45.38)	
No	78.68 (77.17,80.12)	73.31 (71.80,74.77)	67.37 (65.60,69.09)	56.46 (54.62,58.27)	
Diabetes(%)					< 0.0001
Yes	7.50 (6.78,8.30)	8.94 (8.03,9.95)	9.02 (8.14,9.99)	11.92 (10.95,12.96)	
No	92.50 (91.70,93.22)	91.06 (90.05,91.97)	90.98 (90.01,91.86)	88.08 (87.04,89.05)	
Smoked(%)					< 0.0001
Yes	39.15 (37.19,41.15)	42.52 (40.52,44.54)	45.15 (43.46,46.86)	49.33 (47.84,50.83)	
No	60.85 (58.85,62.81)	57.48 (55.46,59.48)	54.85 (53.14,56.54)	50.67 (49.17,52.16)	
Physical Activity(%)					< 0.0001
Never	27.95 (26.14,29.83)	26.86 (25.38,28.40)	24.84 (23.43,26.31)	26.04 (24.57,27.56)	
Moderate	35.21 (33.38,37.09)	30.41 (28.93,31.92)	31.49 (30.05,32.97)	30.69 (29.29,32.12)	
Vigorous	36.84 (35.08,38.64)	42.73 (41.03,44.45)	43.67 (42.15,45.20)	43.28 (41.62,44.94)	
Asthma(%)					0.6941
Yes	85.10 (84.01,86.12)	84.83 (83.65,85.95)	85.28 (83.99,86.48)	85.73 (84.56,86.83)	
No	14.90 (13.88,15.99)	15.17 (14.05,16.35)	14.72 (13.52,16.01)	14.27 (13.17,15.44)	
Coronary Artery Disease(%)					< 0.0001
Yes	2.10 (1.65,2.67)	2.49 (1.99,3.12)	3.88 (3.25,4.62)	4.90 (4.21,5.69)	
No	97.90 (97.33,98.35)	97.51 (96.88,98.01)	96.12 (95.38,96.75)	95.10 (94.31,95.79)	
Cancers(%)					0.0438
Yes	9.90 (9.02,10.87)	8.91 (8.09,9.81)	10.82 (9.75,12.00)	10.13 (9.28,11.04)	
No	90.10 (89.13,90.98)	91.09 (90.19,91.91)	89.18 (88.00,90.25)	89.87 (88.96,90.72)	
Gout(%)					< 0.0001
Yes	1.88 (1.49,2.37)	2.08 (1.65,2.61)	3.15 (2.68,3.70)	8.26 (7.42,9.19)	
No	98.10 (97.61,98.49)	97.83 (97.28,98.28)	96.82 (96.26,97.29)	91.67 (90.73,92.52)	
PIR(%)					0.0285
<1.3	21.06 (19.46,22.76)	20.04 (18.53,21.64)	19.89 (18.39,21.49)	18.78 (17.52,20.10)	
≥ 1.3<3.5	32.10 (30.57,33.67)	33.48 (31.71,35.30)	31.90 (30.03,33.83)	33.58 (31.93,35.27)	
≥ 3.5	38.75 (36.48,41.08)	39.41 (37.01,41.87)	41.35 (39.04,43.70)	40.46 (38.25,42.71)	
Unclear	8.08 (7.12,9.16)	7.08 (6.21,8.05)	6.86 (5.99,7.84)	7.18 (6.31,8.16)	
Total Kcal(%)					< 0.0001
Lower	46.93 (45.16,48.71)	41.67 (40.00,43.37)	36.28 (34.64,37.94)	32.69 (31.41,34.01)	
Higher	38.42 (36.72,40.15)	43.61 (41.86,45.38)	49.03 (47.21,50.85)	52.51 (50.89,54.12)	
Unclear	14.65 (13.28,16.13)	14.71 (13.44,16.09)	14.70 (13.46,16.02)	14.80 (13.72,15.94)	
Total Sugar(%)					0.0279
Lower	37.67 (36.14,39.22)	36.23 (34.53,37.96)	36.39 (34.96,37.85)	35.79 (34.17,37.44)	
Higher	34.64 (33.18,36.12)	37.86 (36.08,39.67)	38.07 (36.48,39.67)	38.07 (36.58,39.58)	
Unclear	27.69 (26.38,29.05)	25.91 (24.19,27.71)	25.54 (24.29,26.83)	26.14 (24.68,27.65)	
Total Water(%)					< 0.0001
Lower	45.69 (44.05,47.35)	39.62 (37.98,41.28)	36.36 (34.75,37.99)	33.87 (32.42,35.35)	
Higher	39.66 (37.96,41.38)	45.67 (44.05,47.30)	48.95 (47.11,50.78)	51.33 (49.74,52.93)	
Unclear	14.65 (13.28,16.13)	14.71 (13.44,16.09)	14.70 (13.46,16.02)	14.80 (13.72,15.94)	
Total Fat(%)					< 0.0001
Lower	45.69 (44.05,47.35)	39.62 (37.98,41.28)	36.36 (34.75,37.99)	33.87 (32.42,35.35)	
Higher	39.66 (37.96,41.38)	45.67 (44.05,47.30)	48.95 (47.11,50.78)	51.33 (49.74,52.93)	
Unclear	14.65 (13.28,16.13)	14.71 (13.44,16.09)	14.70 (13.46,16.02)	14.80 (13.72,15.94)	

For continuous variables: survey-weighted mean (95% CI), P-value was by survey-weighted linear regression (svyglm)

For categorical variables: survey-weighted percentage (95% CI), P-value was by survey-weighted Chi-square test (svytable)

BMI: body mass index; BRI: body roundness index; WWI: weigh adjusted waist index; PIR:ratio of family income to poverty

**Table 2:** Weighted multiple linear regression analysis of the obesity index on serum urate levels

Characteristic	Model 1 β(95%CI)	Model 2 β(95%CI)	Model 3 β(95%CI)
BMI	0.05 (0.05, 0.06)	0.06 (0.06, 0.06)	0.06 (0.05, 0.06)
Tertiles of BMI			
Lower(13.18–25.69)	0	0	0
Middle(25.70-30.88)	0.54 (0.50, 0.58)	0.45 (0.41, 0.48)	0.39 (0.35, 0.42)
Higher(30.89–84.87)	0.85 (0.81, 0.89)	0.93 (0.89, 0.96)	0.84 (0.80, 0.88)
P for trend	< 0.001	< 0.001	< 0.001
WWI Index	0.20 (0.18, 0.22)	0.40 (0.38, 0.42)	0.35 (0.33, 0.37)
Tertiles of WWI			
Lower(8.11–10.70)	0	0	0
Middle(10.71–11.44)	0.23 (0.19, 0.27)	0.33 (0.30, 0.37)	0.27 (0.24, 0.31)
Higher(11.44–15.39)	0.40 (0.36, 0.44)	0.68 (0.64, 0.72)	0.58 (0.54, 0.63)
P for trend	< 0.001	< 0.001	< 0.001
BRI Index	0.14 (0.13, 0.14)	0.18 (0.17, 0.18)	0.17 (0.16, 0.17)
Tertiles of BRI			
Lower(1.05–4.32)	0	0	0
Middle(4.32–6.14)	0.46 (0.42, 0.50)	0.45 (0.42, 0.49)	0.39 (0.35, 0.42)
Higher(6.14–23.48)	0.77 (0.73, 0.81)	0.95 (0.91, 0.99)	0.87 (0.83, 0.90)
P for trend	< 0.001	< 0.001	< 0.001

Model 1 was adjusted for no covariates;

Model 2 was adjusted for age,gender,race,marital status and education;

Model3 was adjusted for covariates in Model 2 + diabetes,blood pressure,PIR,total water,total kcal,total sugar,total fat,smoked,physical activity,alcohol use,serum cholesterol,kidney stone,coronary artery disease,serum glucose,asthma,serum triglycerides and cancers were adjusted

BMI: body mass index; BRI: body roundness index; WWI: weigh adjusted waist index;

**Table 3:** Weighted multiple logistic regression analysis of the effect of obesity index on the prevalence of hyperuricemia

Characteristic	Model 1 OR(95%CI)	Model 2 OR(95%CI)	Model 3 OR(95%CI)
BMI	1.08 (1.08, 1.09)	1.09 (1.09, 1.10)	1.08 (1.08, 1.09)
Tertiles of BMI			
Lower(13.18–25.69)	1	1	1
Middle(25.70-30.88)	2.15 (1.98, 2.34)	2.11 (1.94, 2.30)	1.90 (1.75, 2.07)
Higher(30.89–84.87)	3.86 (3.57, 4.17)	4.20 (3.87, 4.56)	3.57 (3.27, 3.88)
P for trend	< 0.001	< 0.001	< 0.001
WWI Index	1.68 (1.62, 1.74)	1.86 (1.78, 1.94)	1.72 (1.64, 1.80)
Tertiles of WWI			
Lower(8.11–10.70)	1	1	1
Middle(10.71–11.44)	1.76 (1.63, 1.90)	1.82 (1.68, 1.98)	1.63 (1.50, 1.77)
Higher(11.44–15.39)	2.70 (2.50, 2.91)	2.94 (2.69, 3.20)	2.45 (2.24, 2.69)
P for trend	< 0.001	< 0.001	< 0.001
BRI Index	1.26 (1.24, 1.27)	1.29 (1.27, 1.30)	1.26 (1.25, 1.28)
Tertiles of BRI			
Lower(1.05–4.32)	1	1	1
Middle(4.32–6.14)	2.27 (2.09, 2.47)	2.24 (2.05, 2.44)	1.99 (1.82, 2.18)
Higher(6.14–23.48)	4.16 (3.84, 4.50)	4.46 (4.10, 4.86)	3.77 (3.45, 4.12)
P for trend	< 0.001	< 0.001	< 0.001

Model 1 was adjusted for no covariates;

Model 2 was adjusted for age,gender,race,marital status and education;

Model3 was adjusted for covariates in Model 2 + diabetes,blood pressure,PIR,total water,total kcal,total sugar,total fat,smoked,physical activity,alcohol use,serum cholesterol,kidney stone,coronary artery disease,serum glucose,asthma,serum triglycerides and cancers were adjusted

BMI: body mass index; BRI: body roundness index; WWI: weigh adjusted waist index;

**Table 4:** Diagnostic efficacy of ROC analysis of obesity-related indices for hyperuricemia

Test	ROC area(AUC)	95%CI low	95%CI upp	Best threshold	Specificity	Sensitivity
BMI	0.6635	0.656	0.6711	27.535	0.5114	0.7307
WWI	0.6222	0.6144	0.63	10.9142	0.4643	0.7115
BRI	0.6692	0.6617	0.6766	5.4202	0.5967	0.6498

BMI: body mass index; BRI: body roundness index; WWI: weigh adjusted waist index;

**Table 5:** Weighted multiple logistic regression analysis of the effect of obesity index on the prevalence of gout

Characteristic	Model 1 OR(95%CI)	Model 2 OR(95%CI)	Model 3 OR(95%CI)
BMI	1.05 (1.04, 1.05)	1.07 (1.06, 1.08)	1.05 (1.04, 1.06)
Tertiles of BMI			
Lower(13.18–25.69)	1	1	1
Middle(25.70-30.88)	1.77 (1.51, 2.07)	1.54 (1.31, 1.82)	1.33 (1.12, 1.57)
Higher(30.89–84.87)	2.57 (2.22, 2.99)	3.01 (2.58, 3.53)	2.16 (1.83, 2.54)
P for trend	< 0.001	< 0.001	< 0.001
WWI Index	1.80 (1.69, 1.92)	1.58 (1.45, 1.72)	1.31 (1.19, 1.43)
Tertiles of WWI			
Lower(8.11–10.70)	1	1	1
Middle(10.71–11.44)	2.36 (1.99, 2.80)	1.65 (1.38, 1.97)	1.38 (1.15, 1.65)
Higher(11.44–15.39)	3.64 (3.09, 4.27)	2.24 (1.86, 2.69)	1.56 (1.29, 1.88)
P for trend	< 0.001	< 0.001	< 0.001
BRI Index	1.18 (1.15, 1.20)	1.21 (1.19, 1.24)	1.15 (1.12, 1.18)
Tertiles of BRI			
Lower(1.05–4.32)	1	1	1
Middle(4.32–6.14)	2.18 (1.85, 2.58)	1.57 (1.32, 1.87)	1.32 (1.10, 1.57)
Higher(6.14–23.48)	3.47 (2.96, 4.06)	2.93 (2.48, 3.46)	2.02 (1.70, 2.41)
P for trend	< 0.001	< 0.001	< 0.001

Model 1 was adjusted for no covariates;

Model 2 was adjusted for age,gender,race,marital status and education;

Model3 was adjusted for covariates in Model 2 + diabetes,blood pressure,PIR,total water,total kcal,total sugar,total fat,smoked,physical activity,alcohol use,serum cholesterol,kidney stone,coronary artery disease,serum glucose,asthma,serum triglycerides and cancers were adjusted

BMI: body mass index; BRI: body roundness index; WWI: weigh adjusted waist index;

**Table 6:** Diagnostic efficacy of ROC analysis of obesity-related indices for gout

Test	ROC area(AUC)	95%CI low	95%CI upp	Best threshold	Specificity	Sensitivity
BMI	0.6112	0.5963	0.626	27.895	0.4922	0.6873
WWI	0.6457	0.6321	0.6593	11.1863	0.5609	0.6609
BRI	0.6436	0.6295	0.6577	5.2234	0.5226	0.7092

BMI: body mass index; BRI: body roundness index; WWI: weigh adjusted waist index;

### Electronic supplementary material

Below is the link to the electronic supplementary material.

Supplementary Material 1

## Data Availability

The data used or analyzed in this study are available from the corresponding author upon reasonable request.
