# Supplementary material for: Relationship between gout, hyperuricemia, and obesity—does central obesity play a significant role?—a study based on the NHANES database
Source: Diabetol Metab Syndr. 2024 Jan 22;16:24. doi: 10.1186/s13098-024-01268-1 (PMC10804703; doi:10.1186/s13098-024-01268-1)
Supplement: Supplementary file 1 — Supplementary Material 1 [file 13098_2024_1268_MOESM1_ESM.docx]

**Supplementary Table 1.**Weighted multiple logistic regression analysis of the effect of obesity index on the prevalence of hyperuricemia(urate>7mg/dL).

| **Characteristic** | **Model 1 OR(95%CI)** | **Model 2 OR(95%CI)** | **Model 3 OR(95%CI)** |
| --- | --- | --- | --- |
| BMI | 1.06 (1.06, 1.07) | 1.09 (1.08, 1.09) | 1.08 (1.08, 1.09) |
| WWI Index | 1.38 (1.30, 1.41) | 1.88 (1.79, 1.98) | 1.74 (1.64, 1.83) |
| BRI Index | 1.18(1.17, 1.20) | 1.28 (1.26, 1.29) | 1.26 (1.24, 1.28) |

Model 1 was adjusted for no covariates;

Model 2 was adjusted for age,gender,race,marital status and education;

Model3 was adjusted for covariates in Model 2+diabetes,blood pressure,PIR,total water,total kcal,total sugar,total fat,smoked,physical activity,alcohol use,serum cholesterol,kidney stone,coronary artery disease,serum glucose,asthma,serum triglycerides and cancers were adjusted.

BMI: body mass index; BRI: body roundness index; WWI: weigh adjusted waist index;
